# Investigation of process parameters in orthogonal cutting using finite element approaches

**DOI:** 10.1016/j.heliyon.2020.e05498

**Published:** 2020-11-14

**Authors:** H.A. Soliman, A.Y. Shash, T.M. El Hossainy, M. Abd-Rabou

**Affiliations:** a Material Science and Engineering Department, Egypt-Japan University of Science and Technology, 21934, Alexandria, Egypt; b Manufacturing Engineering and Production Technology Dept., Modern Academy for Engineering and Technology, 11585, Cairo, Egypt; c Mechanical Design and Production Engineering Dept., Faculty of Engineering, Cairo University, 12316, Giza, Egypt; d Faculty of Engineering and Materials Science, German University in Cairo, 11432 Cairo, Egypt; e Zewail City of Science and Technology, 12578, Giza, Egypt

**Keywords:** Industrial engineering, Materials science, Mechanical engineering, Manufacturing engineering, Machine design, Machining, Orthogonal cutting, Finite element analysis, Lagrangian method, Arbitrary Lagranigan-Eulerian method, Johnson-cook model

## Abstract

The cutting force in orthogonal cutting of steel AISI 1045 was predicted by applying 2D finite element analysis (FEA) using two methods; (i) Lagrangian (LAG) and (ii) Arbitrary Lagrangian Eulerian (ALE). Johnson-Cook (J-C) models were used for defining plastic and failure properties of simulated materials. The predicted force was validated experimentally by using dynamometer. Comparison held between the simulation methods and experimental work in terms of results accuracy, reading stability, and chip morphology. Furthermore, this study adopted new modeling idea to control the excessive distortion of mesh elements along chip separation line by defining nearly zero damage criterion for these elements. The results demonstrated that LAG and ALE methods could predict the cutting force but with different accuracy, as LAG and ALE results deviated from experimental results with minimum error percentage 3.6% and 0.14% respectively. As well, ALE method showed stable force readings and continues smooth chip during simulation, while LAG method showed unstable force readings and discontinuous realistic chip.

## 1 Introduction

Machining processes and their relevant subjects have always great interest for researchers due to their vital role in the industry and economy. Mamalisa et al. (2001) stated that researchers initially studied the machining processes in its simplified orthogonal cutting in 1940. Since that time, metal cutting researchers have established the theories of orthogonal cutting relating chip formation, cutting temperature, tool wear, cutting forces, etc. Admittedly, cutting force is one of the most significant parameters in machining system due to its great influence on tool wear, workpiece dimensions and machine power. Basically, cutting force is one of the three force components resulted from the machining action, which is defined as the component of the total force acting along the direction of cutting speed. Cutting force can be, roughly, calculated by mathematical laws or, exactly, measured by dynamometer device. Cutting force prediction has fundamental importance for improving tool design and optimization of cutting conditions. As well, it has economical influences in terms of tool wear prediction and, consequently, tool life and cost estimation. Although many approaches have been presented for modeling metal cutting, finite element analysis (FEA) is still one of the most reliable and accurate methods. Ali et al. (2017) presented methodology for predicting tool wear in orthogonal cutting process by simulating the contact stresses on the tool face using FEA. This study proved the ability of FEA in modeling the metal cutting processes.

Starting from material modeling, Chen et al. (2019) confirmed that the material constitutive models influence the FEA results. Thus, their study demonstrated that Johnson-Cook (J-C) model is suitable for defining material properties in orthogonal cutting simulation. Also, Shah et al. (2019) applied J-C model for defining the simulated materials, and the FEA results showed realistic serrated chip similar to experiments. Furthermore, Kilikaslany (2009) held comparative study between four material models, Oxley, Zeirilli and Armstrong, and Johnson-Cook for modeling orthogonal cutting process and he proved that Johnson cook model can be used to predict the shear angles and cutting forces with high accuracy. Hence, it can be concluded that depending on the applied material model, the cutting process can be simulated ideally and the cutting parameters can be predicted accurately. Consequently, applying J-C material model in orthogonal cutting simulation promotes realiable simulation with accurate results. For instance, Kara et al. (2016) were able to predict cutting temperatures in orthogonal cutting process by applying FEA and using J-C material model, and the simulation results showed agreement with experiments. Also, Malakizadi et al. (2016) predicted flank wear by using J-C material model in their FEA, and the predicted flank wear rate agreed with most of the experimental results.

**Figure 1:**
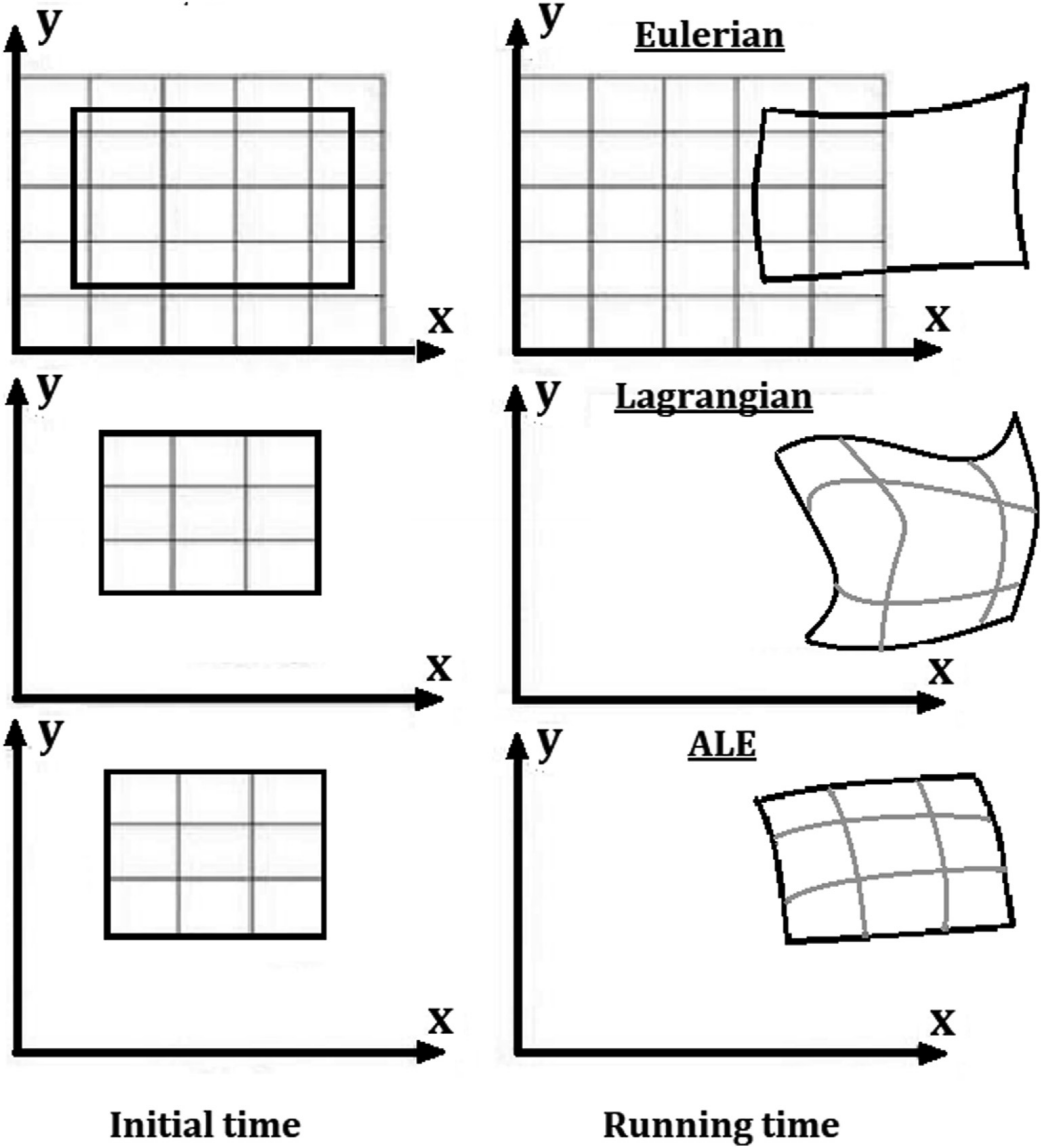
Eulerian, Lagrangian, and ALE meshing.

Taking a deeper sight into FEA methods, Soliman et al. (2020) explained that the motion of chip removal can be simulated using FEA by three methods: Eulerian (EUL), Lagrangian (LAG), and Arbitrary Lagrangian Eulerian (ALE) method. As shown in Figure 1 EUL, the mesh points are fixed but the material points move freely without being constrained with the mesh grid. On the contrary in Figure 1 LAG, the mesh points move in conjunction with the material points. Finally In Figure 1 ALE, the material and mesh points move together in conservative way in order to improve the distance ratio between elements during motion without destructing the mesh grading. So, ALE method combines characteristics from EUL and LAG. Grissa et al. (2018) applied the three methods to predict residual stresses in orthogonal cutting simulation and they concluded that LAG and ALE methods were more realistic for chip removal simulation and close to experimental results than EUL method. However, this study did not give sufficient comparison of these two methods in terms of cutting force prediction.

Excessive distortion of mesh elements is one of the destructive problems in modeling metal cutting processes. Basically, elements are susceptible to be excessively distorted under high compression loads and/or high strain rates. Thus, FEA programs and researchers try to avoid this problem by applying different techniques such as distortion control, adaptive meshing, particle finite element method (PFEM), etc. Finite element programs, such as Abaqus, offer distortion control option by defining distortion length ratio which prevents elements to be excessively distorted by exceeding this ratio. Also, adaptive meshing is another method which is widely used in metal cutting processes simulation for avoiding excessive distortion. Adaptive meshing occurs after number of simulation time increments, as the current mesh is remeshed by reallocating the nodes in adapted positions to control any distortion. Wan et al. (2019) adopted this method during the FEA of micro milling process to predict the cutting forces, and it helped to control element distortion in the cutting area and consequently the results were accurate and agreed with experiments. .On the other hand, Rodríguez et al. (2017) presented a numerical technique to avoid element excessive distortion, which is called particle finite element simulation. This technique applies sequential and repetitive steps of removing mesh particles, elements, depending on distance and error estimator, defined by the user, and consequently the distorted boundary of the part is refined after each deletion step.

It is worth noting that the above modelling approaches were not the only ones that deal with the large deformation and excessive distortion problems, as there are other approaches were presented in the literature to solve this problem such as Coupled Eulerian-Lagrangian (CEL) formulation and meshless free methods. Ducobu et al. (2017) applied CEL approach in 3D orthogonal cutting simulation and the force results showed error percent with experiemnts less than 10%. Kaselouris et al. (2017) adopted Smoothed Particle Hydrodynamics (SPH) meshless method for modeling orthogonal cutting process. Their work showed that SPH and mesh free methods are prominent computational tools when it comes to handle problems of severe deformation, however they are not the right choice if the distortions could be controlled since they are more computationally expensive than FEM. Other researchers tried to improve the material model itself such as Calamaz et al. (2008) who proposed material model named TANH (Hyperbolic TANgent) by adding new term to the J-C law in order to consider the strain softening effect. On the other side, other researchers implemented finite element analysis to predict the change in microstructure during the orthogonal cutting such as Jafarian et al. (2014) who simulated the dynamic recrystallization and, consequently, predicted the grain refinement and hardness variation during the orthogonal cutting of Inconel 718 alloy. by implementing user subroutine in FE code taking into account the microstructure of the material using Zener–Hollomon and Hall–Petch equations.

**Figure 2:**
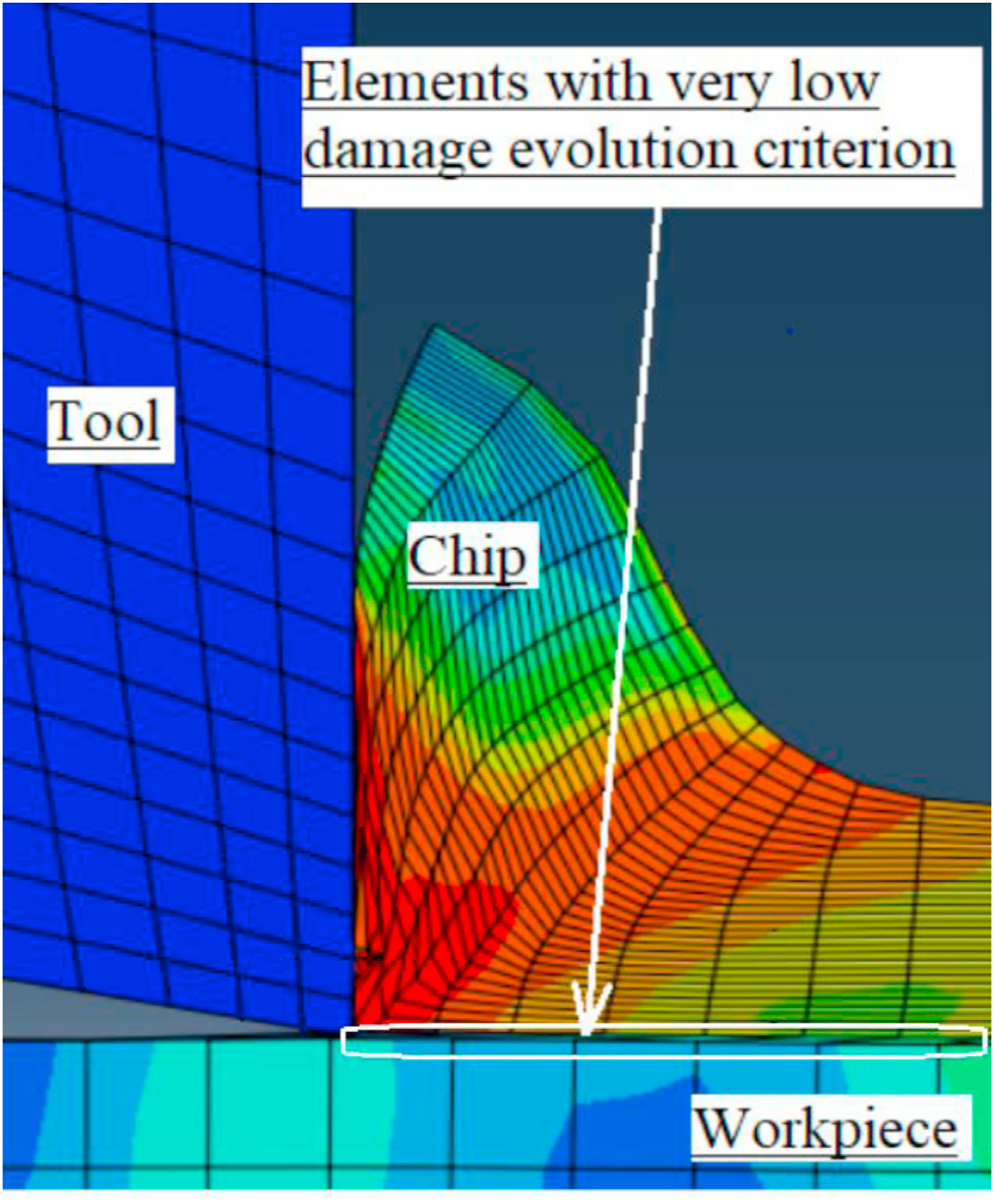
Controlling element distortion towards sharp tool-tip.

Finally, although, many ideas and techniques were presented in the literature for distortion control, there is some shortage of using finite element modeling ideas in terms of defining nearly zero damage evolution criterion for elements susceptible to excessive distortion towards the sharp edge of tool-tip as shown in Figure 2. As these elements will be deleted from simulation whenever high contact occurred between tool and workpiece without conflicting the chip form or FEA results. Also, as aforementioned that there is research gap in comparing Lagrangian and Arbitrary Lagrangian Eulerian approaches,specially, for cutting force modeling. Although, recently researchers such as Grissa et al. (2018) tried to present comparative study between these approaches, but their study concentrated on residual stress rather than cutting force modeling. So, this current work presents comparative study concentrating on cutting forces modeling under different cutting conditions with experimental validation.

## 2 Finite element simulation

The simulation work included the modeling steps for orthogonal cutting of steel AISI 1045 workpiece with tungsten carbide (WC) tip by using ABAQUS/CAE at different feed rates [0.1, 0.2, 0.3, 0.4 and 0.48 mm/rev] in order to predict cutting force.

### 2.1 Model formulation

**Figure 3:**
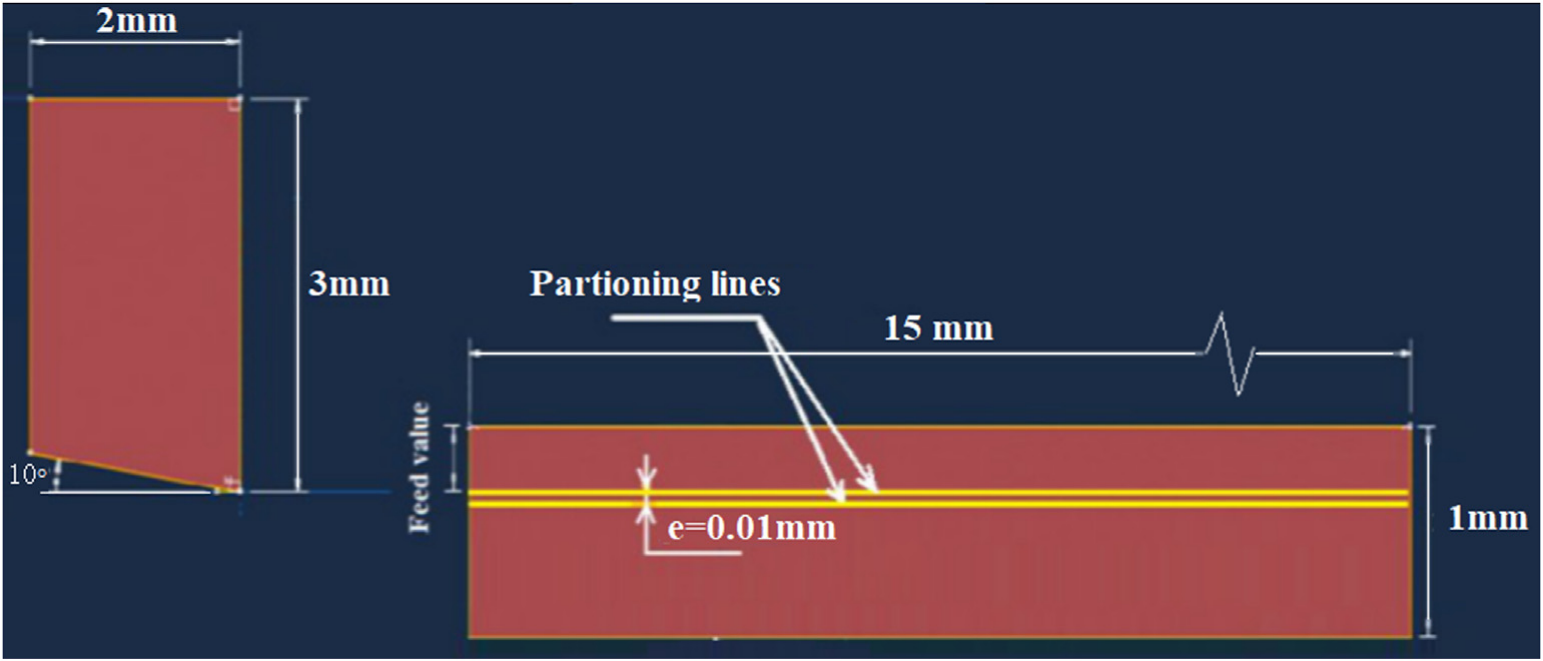
Tool and workpiece model dimensions.

The carbide tip and steel workpiece were simulated as two dimensional deformable models. As shown in Figure 3, small dimensions used to represent the carbide tip and workpiece models in order to reduce FEA running time. In workpiece model, a partition line was modeled with depth equal the feed value, as well another one was created below it with distance (e) = 0.01 mm. The first line defined the chip separation line through workpiece and the second line was modeled to form a very thin damage area between the two lines that contained elements with very low damage criterion, nearly zero,. The value of (e) was chosen in order to give reliable chip form and accurate results. It is worth noting that there was a relation between (e) and the mesh element size in the chip area. In other words, the best value for (e) is to be equal or less than the mesh element size chosen for chip modeling. This idea can reduce the excessive distortion of mesh elements that may be caused due to the high penetration of sharp tool tip in workpiece model.

**Figure 4:**
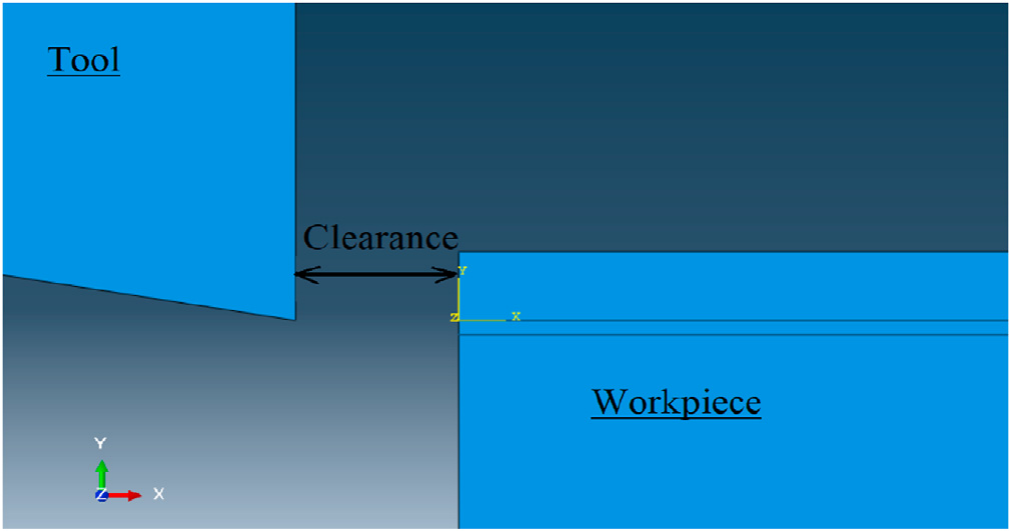
Clearance between tool and workpiece.

**Figure 5:**
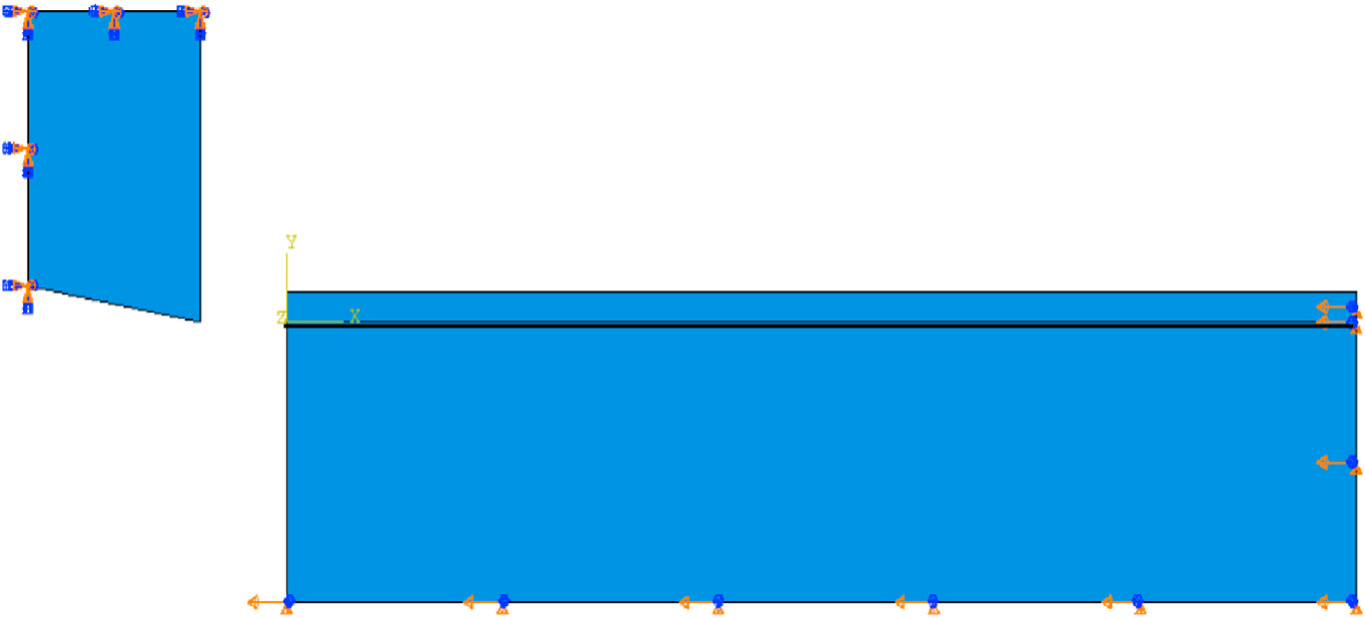
Model boundary condition.

**Figure 6:**
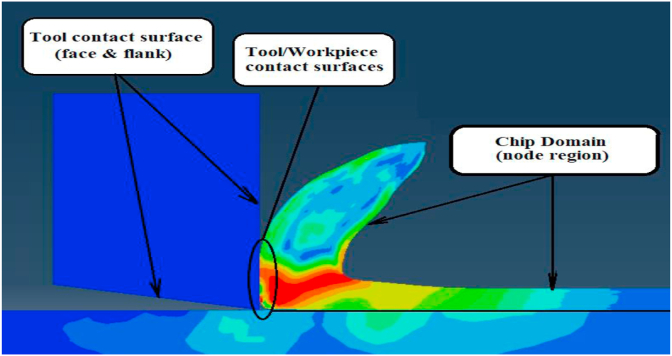
Model contact pairs.

For assembly, the tool-tip and workpiece were assembled with clearance between them as shown in Figure 4 to avoid inertia effect when contact is occurred between surfaces in the beginning of motion. Then, the simulation step time was calculated by knowing the model displacement and velocity values. As well, the cutting velocity values were set as boundary condition for workpiece model, while the tool model defined as fixed in position, Figure 5. Furthermore, friction contact was defined between the tool and workpiece surfaces as shown in Figure 6.

### 2.2 Material modeling

The tool and workpiece material properties were defined for FEA program. Firstly, the tool was defined as tungsten carbide (WC) and its properties are shown in Table 1:

**Table 1:** WC properties [Soliman et al. (2020)].

Density [g/cm^3]^	Young's Modulus [GPa]	Poisson's ratio
15.63	550	0.234

Salvatore et al. (2013) mentioned the tungsten carbide properties for accurate FEA modeling in terms of stress- strain data for plastic properties and Johnson-Cook parameters for damage properties as shown in Tables 2 and 3, respectively.

**Table 2:** Stress-strain data for WC [Salvatore et al. (2013)].

Effective plain strain	Yield Stress [MPa]
0	419.53
0.000263	1696.9
0.00097364	2675.5
0.002107	3376.5
0.003574	3858.2
0.005287	4178
0.007176	4382.2
0.00919	4504.8
0.01129	4570.1
0.013452	4595.5
0.014279	4597.3
0.1	4597.3

**Table 3:** Johnson-Cook damage parameters for WC [Salvatore et al. (2013)].

d_1_	d_2_	d_3_	d_4_	d_5_
0	0.01072	-1.669	0	0

Secondly, the workpiece material was defined as steel AISI 1045 and its properties are shown in Table 4:

**Table 4:** Steel AISI 1045 properties [Ślusarczyk and Franczyk, 2019].

Density [g/cm^3]^	Young's Modulus [GPa]	Poisson's ratio
7.85	210	0.269

Duan et al. (2011) stated the AISI 1045 properties for finite element modeling by defining Johnson-Cook plastic and damage parameters as shown in Tables 5 and 6, respectively.

• Johnson-Cook Damage Model [Table 6].

**Table 5:** Johnson-Cook plastic parameters for Steel AISI 1045 [Duan et al. (2011)].

A [MPa]	B [MPa]	m	c	n	Reference strain rate
553	600	1	0.0134	0.234	0.001

**Table 6:** Johnson-Cook damage parameters for Steel AISI 1045 [Duan et al. (2011)].

d_1_	d_2_	d_3_	d_4_	d_5_
0.06	3.31	-1.96	0.0018	0.58

**Figure 7:**
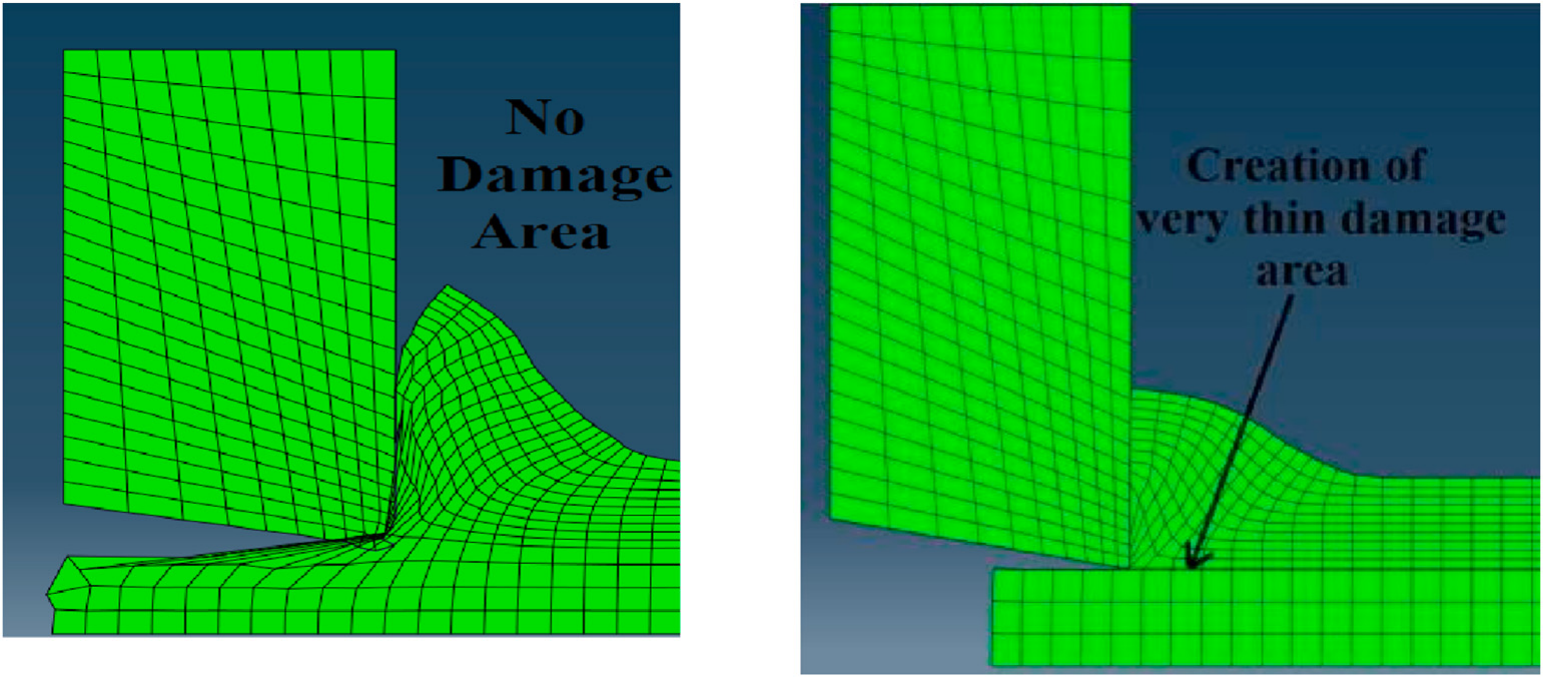
The effect of thin damage area on mesh distortion.

Finally, The material properties for the very thin damage area (e = 0.01 mm), between the two partitioning lines, were defined as AISI 1045 but with very low damage criterion. This means that the element will be fail if its strain equal or less than the element size, which promotes fast deletion for the elements before any excessive distortion as shown in Figure 7.

### 2.3 Meshing

**Figure 8:**
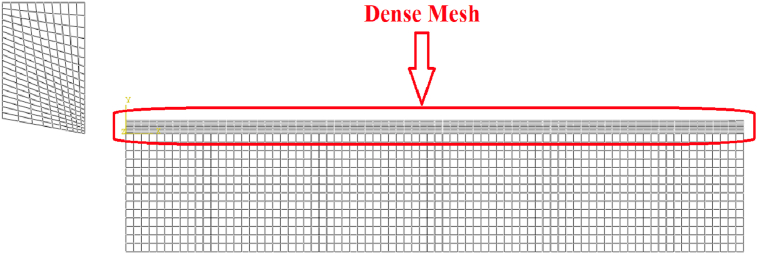
Model mesh generation.

For meshing, four node plane strain element (CPE4R) were assigned for modeling the tool-tip and workpiece models. This type of elements is quadrilateral with automatic hourglass control and reduced integration. The density of mesh elements influences the accuracy of extracted results from simulation analysis. Therefore, all feed rates were simulated with dense mesh in the upper part of workpiece that will be separated later in form of chip after tool penetration, as in Figure 8. That's to model realistic chip form and to enhance results accuracy.

**Figure 9:**
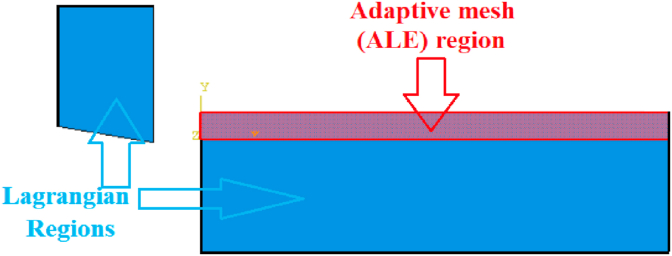
ALE model.

In ALE model, adaptive meshing was necessary to be applied for the upper part of workpiece to control the element distortion in chip form. Figure 9 shows the distribution of adaptive meshing and Lagrangian regions in ALE model. On the other hand, LAG model did not follow adaptive meshing technique.

### 2.4 Cutting force prediction

**Figure 10:**
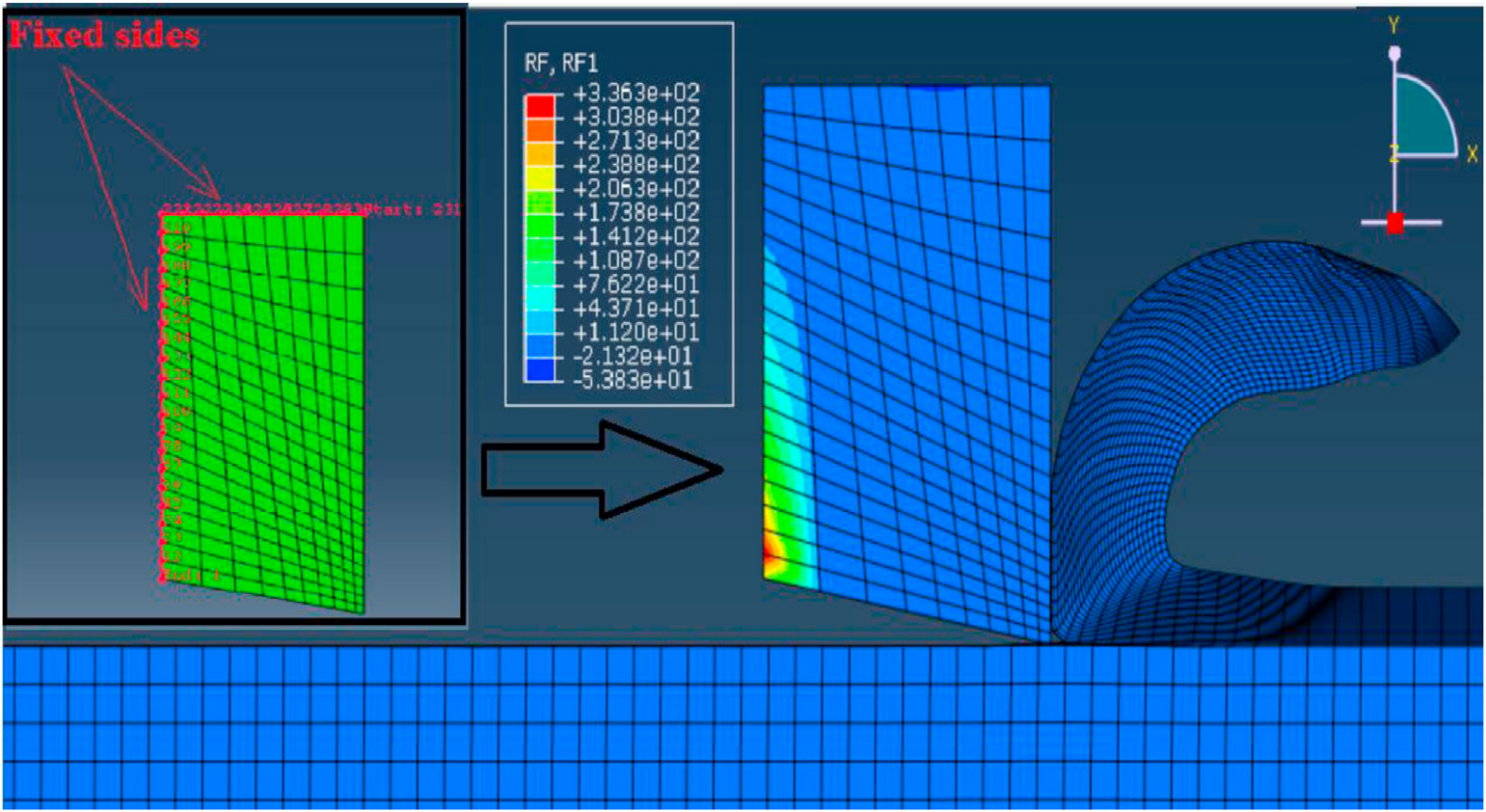
Cutting force prediction.

After the analysis was completed, the reaction forces extracted from the tool model. To predict the cutting forces, a path of nodes was defined on the fixed sides of tool model as shown in Figure 10. Thus, the reaction forces on these nodes, in x direction, were summed for each step during the simulation time, then an average value was calculated for all the steps to predict the cutting force at each feed rate.

## 3 Experimental work

Experimental work was held in two dependant stages; Firstly, the dynamometer was calibrated to be used later for indicating the forces during the machining process. Soliman et al. (2020) calibrated the dynamometer device used in the current study and this is a complementary work. Secondly, the orthogonal cutting of steel AISI 1045 bars was implemented on center lathe machine by using WC tool-tip. During machining experiments, the cutting velocity (v) was constant [v = 100 m/min] and the depth of cut (t) was also constant [t = 1 mm], while the feed rate (f) was variable [f = 0.1, 0.12, 0.2, 0.3, 0.4 and 0.48 mm/rev.].

## 4 Results & discussion

### 4.1 Experimental results

**Figure 11:**
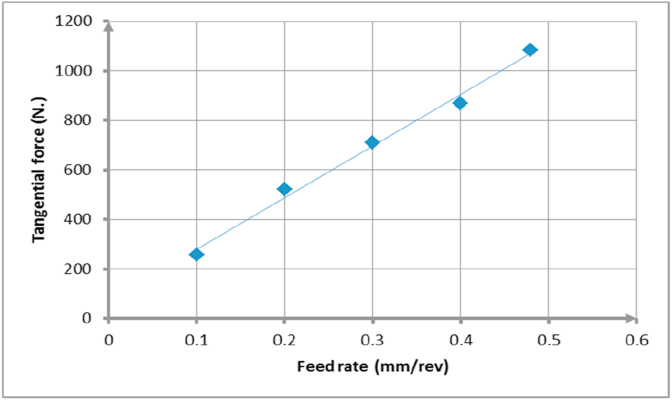
Cutting force with feed values.

Table 7 shows the measured values of cutting forces using dynamometer device. It was demonstrated that there was linear relationship between feed rates and cutting forces values, Figure 11. This relationship was due to the increase in chip volume which consequently increased the contact force on tool face.

**Table 7:** Experimental cutting force values.

Feed rates (mm./rev)	Cutting force (N.)
0.1	256
0.15	336
0.2	523
0.3	709
0.4	869
0.48	1082

### 4.2 Finite element analysis (FEA) results

Table 8 shows the cutting forces extracted from LAG and ALE model at each feed rate compared with the experimental results. As shown from the table, the minimum and maximum error percentages for LAG model were 3.6% and 14.4 %, respectively, while the minimum and maximum error percentages for ALE model were 0.14% and 5.8%, respectively.

**Table 8:** LAG, ALE and experimental force results.

Feed rates (mm./rev.)	LAG cutting force (N.)	ALE cutting force (N.)	Experimental cutting force (N.)	LAG I Error I %	ALE I Error I %
0.1	293	271	256	14.4	5.8
0.2	504	497.5	523	3.6	4.9
0.3	670	710	709	5.5	0.14
0.4	925.5	896.5	869	6.5	3.2
0.48	998	1038	1082	7.7	4.1

The LAG and ALE approaches were validated with experimental results and comparison held between them in terms of three criteria:

• Accuracy
• Stability
• Chip morphology

Firstly, from Table 8, it can be seen that the deviation from experimental force results was lower in ALE than LAG results, which means that ALE approach were more accurate for predicting the cutting force. The reason for accurate ALE results is the concept of its adaptive meshing in terms of avoiding mesh distortion during chip separation that influences the force results. Whenever the mesh distortion increased, the cutting forces increased due to the unreal effect of mesh distortion during simulation and that was the reason for the higher force results of Lagrangian model.

**Figure 12:**
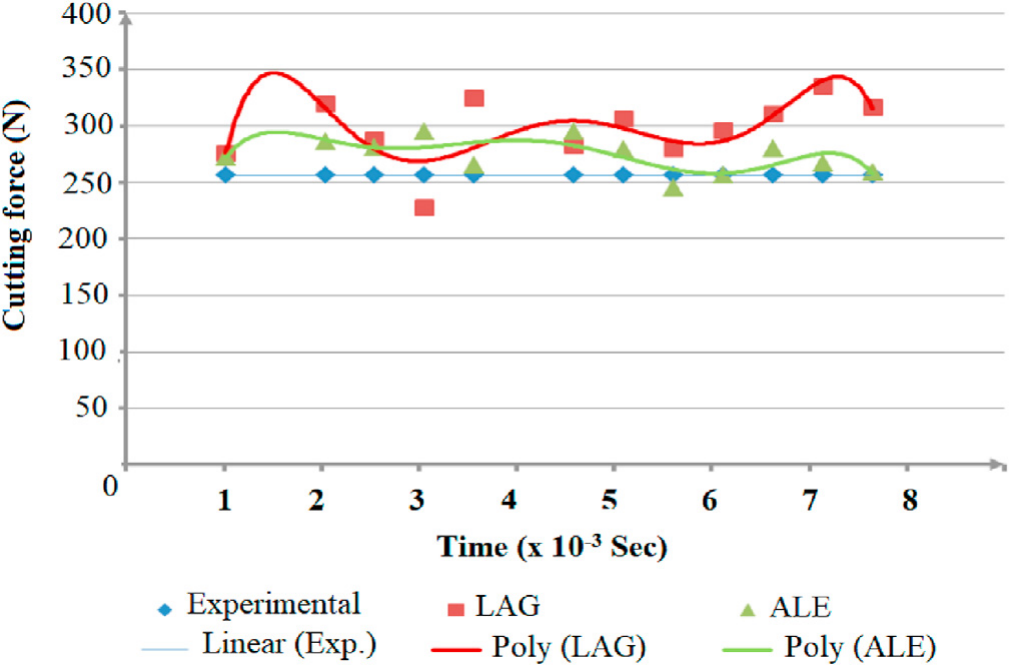
Cutting forces at f = 0.1 m.m./rev.

**Figure 13:**
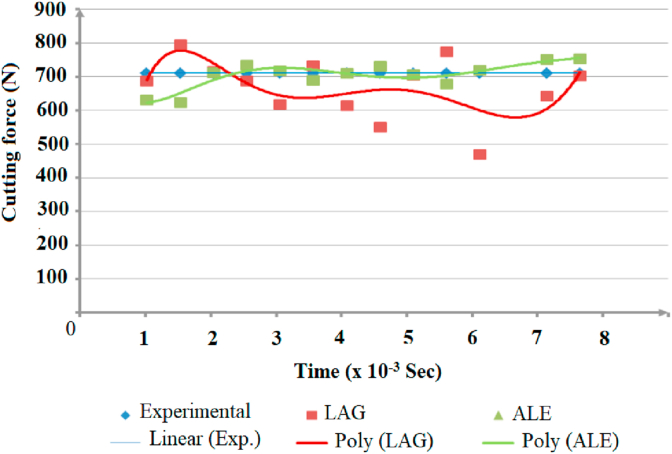
Cutting force at f = 0.3 m.m./rev.

**Figure 14:**
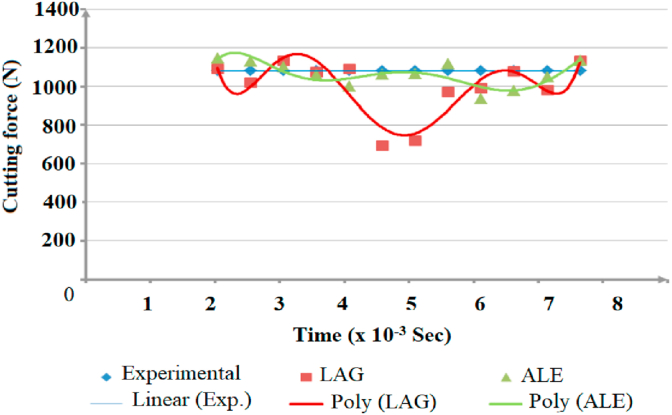
Cutting forces at f = 0.48 m.m./rev.

**Figure 15:**
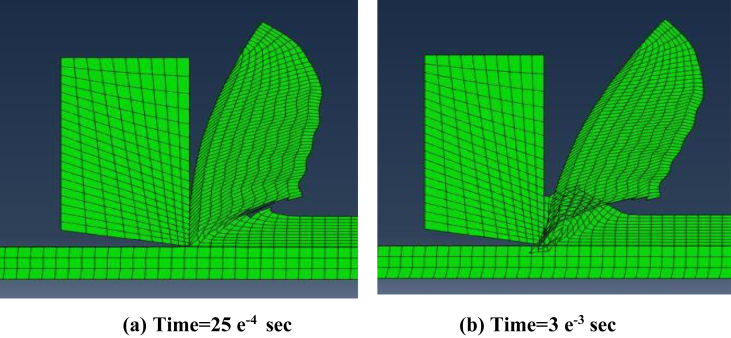
Excessive distortion and chip separation in Lagrangian model.

**Figure 16:**
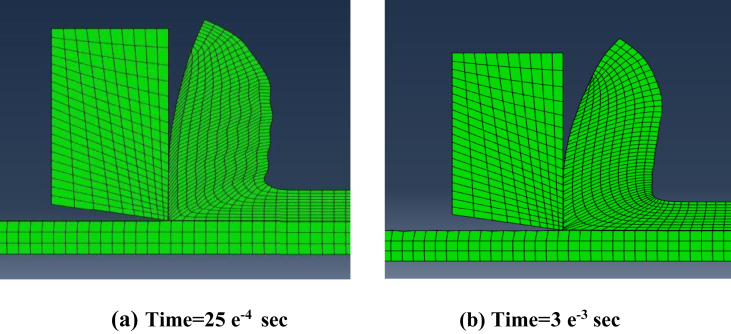
ALE chip morphology.

Secondly, steadiness of readings during simulation process was vital factor in comparing modeling methods. Figures 12, 13, and 14 show the change of the cutting forces during simulation time for LAG, ALE models for three feed rates values (f = 0.1, 0.3 and 0.48). As indicated from the shown figures, ALE was steadier than LAG approach and its readings were closer to experiments. The reason for LAG readings divergence was the accumulation and deletion of the excessive distorted mesh elements towards the tool-tip during its penetration in workpiece. As the cutting forces increased when the distorted mesh elements accumulated, but then decreased sharply when the chip was cut off away. This rise and decline in force readings were repeated along the simulation time. This can be clarified in Figure 15 which shows the propagation of chip separation in Lagrangian model. On the other hand, Figure 16 demonstrates that the adaptive meshing in ALE approach controlled the mesh distortion by conserving the ratio between elements during the remeshing. This, eventually, caused better accuracy and steadiness for ALE readings during the simulation process.

Finally, Figures 15 and 16 also depict the chip form in LAG and ALE simulation. Typically, the uncontrolled excessive mesh distortion is characterized in LAG simulation, while ALE simulation with adaptive meshing conserves the ratio between mesh elements before the incidence of excessive mesh distortion. Thus, ALE model showed smooth chip form during the simulation running time, while LAG model showed distorted and discontinuous chip form.

### 4.3 The advantages of creation the low damage evolution area

**Figure 17:**
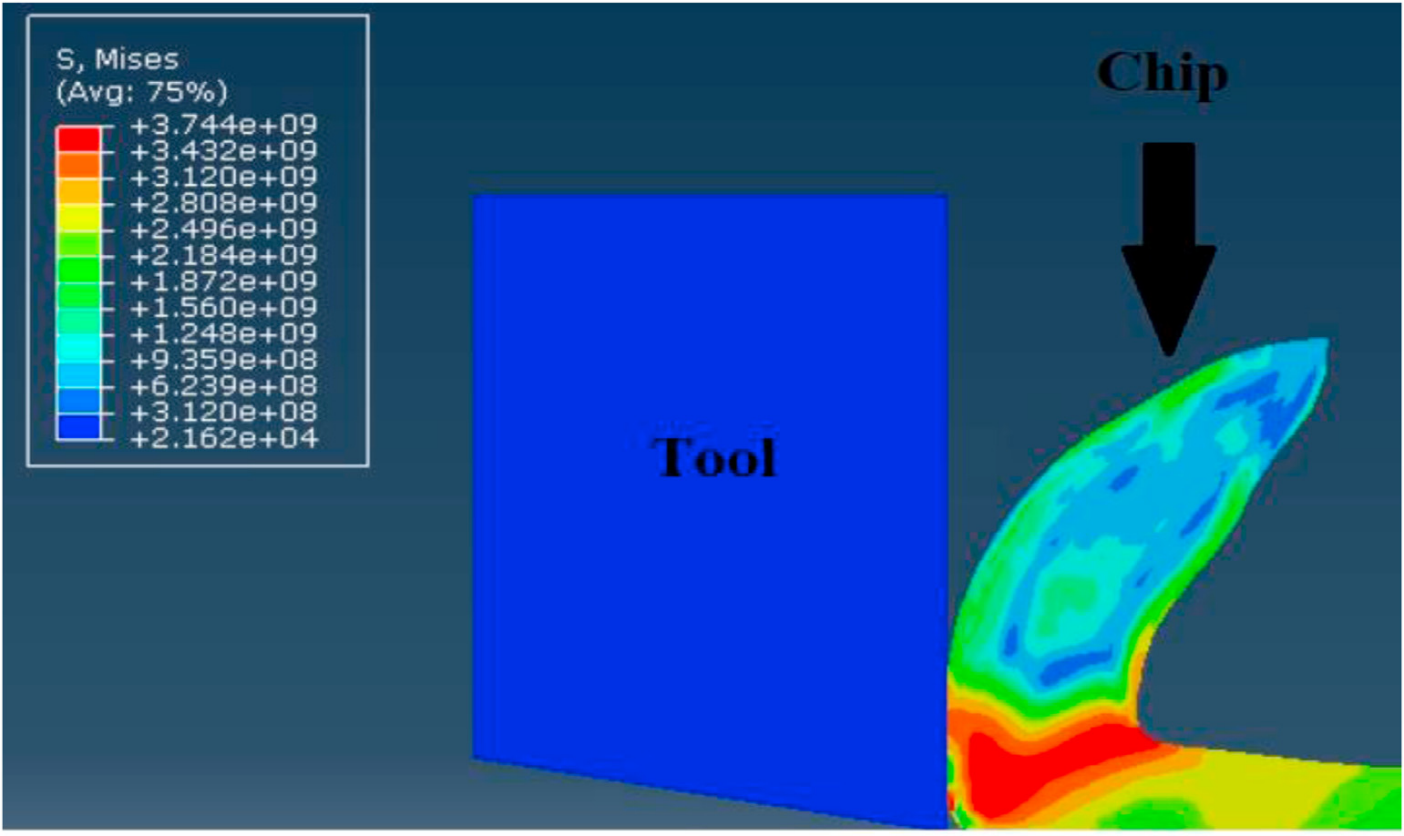
Shear stresses on shear plane at t = 4 e^−3^ sec.

**Figure 18:**
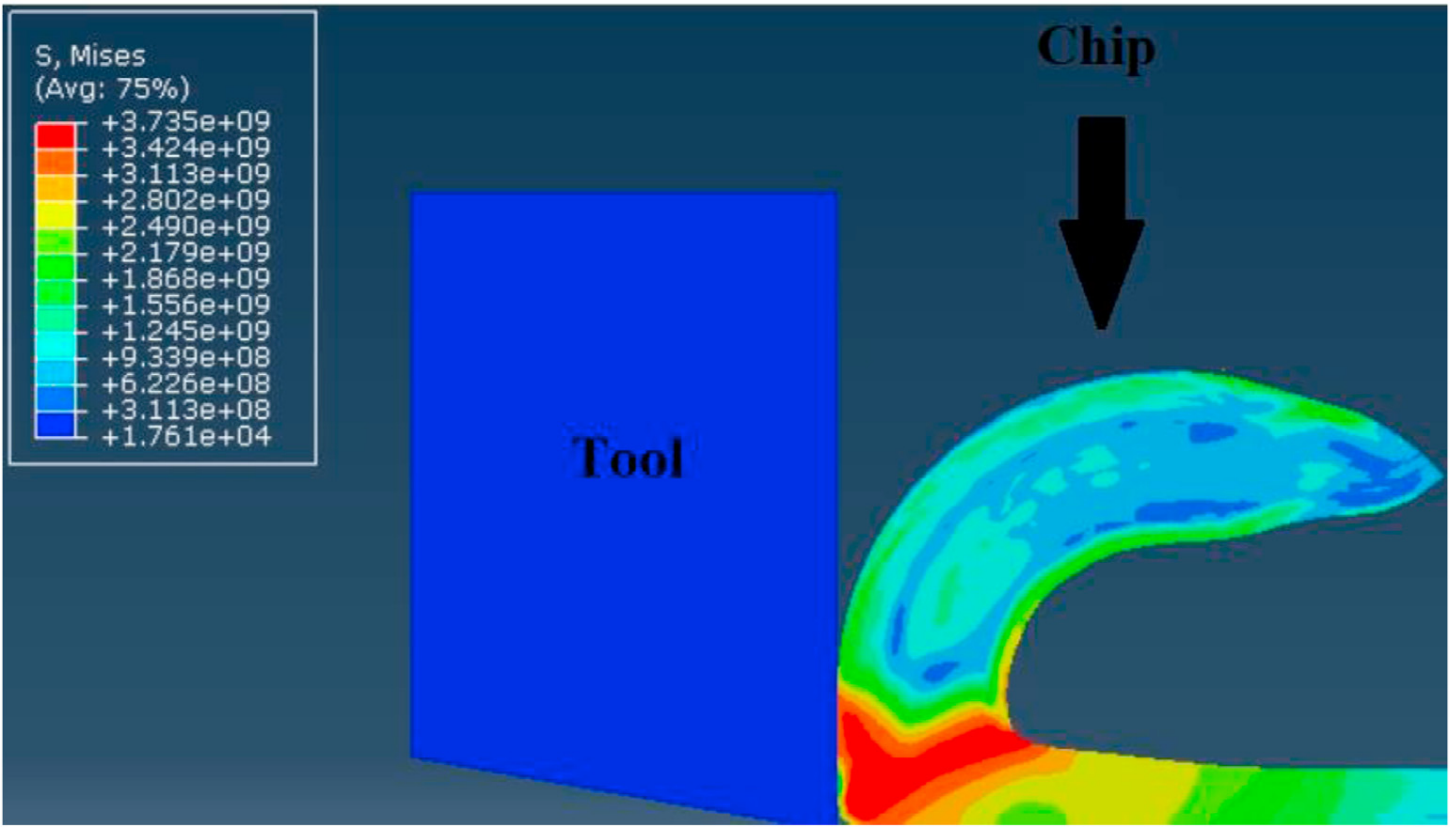
Shear stresses on shear plane at t = 5.6 e^−3^ sec.

**Figure 19:**
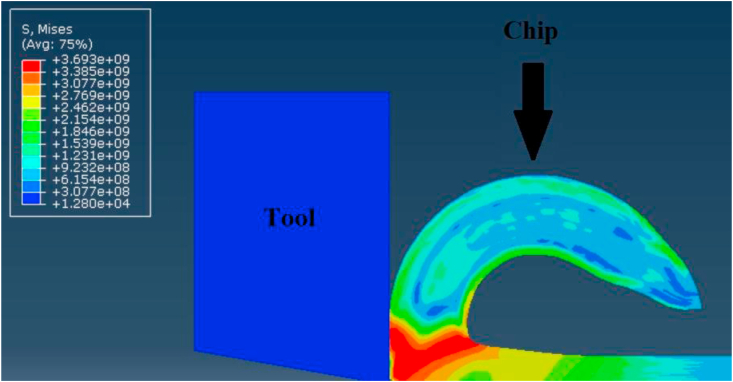
Shear stresses on shear plane at t = 6.6 e^−3^ sec.

Minimization of simulation running time, without conflicting the accuracy of results, was essential criterion to evaluate the efficiency of the used simulation methodology. Thus, the comparison between the two models, with and without thin area of nearly zero damage evolution, demonstrated that creation of this area in the model could reduce the running time to 44 %. As the model with low damage area could cut 5.7 mm from the workpiece in two hours, while the other model could cut only 3.2 mm during the same period and using the same computer hardware capabilities (Core i7, 1.8 GHz and 8 GB of RAM). This reduction in simulation time was in virtue of controlling the excessive distortion by defining very low damage evolution criterion in the region of chip separation, which led to delete the distorted elements fast and reduce the computational capacity of the model analysis. In addition to the previous advantage, this idea enhanced the chip morphology. On the other hand, this distinctive methodology prevented any geometrical interference of the sharp tool model inside the workpiece in order to ensure reliable contact between them. All these advantages had much of a role to play with the minimization of simulation errors in FEA to achieve high prediction for cutting force. Finally, Figures 17, 18, and 19 shows the shear stresses for chip separation zone at different simulation times (t) which proved the reliability and steadiness of results during the simulation process in the virtue of the recommended methodology. It is worth noting that the domain of validity of this model is limited to the case where the feed per tooth is large with respect to the cutting edge radius.

## 5 Conclusions

Finite element analysis (FEA) has demonstrated its capability in metal cutting simulation. This study has proved the accuracy and reliability of FEA in orthogonal cutting simulation using two approaches; LAG and ALE. This paper has depicted some highlights which can be summarized as following:

1) Agreement of using Johnson-Cook (J-C) constitutive models for material modeling in FEA, as the simulation results were validated experimentally.
2) Modeling by using finite element programs allows creation of partition lines inside the model which was utilized in this paper to increase the accuracy of results. Creation of partition lines, inside the workpiece, enhances the chip separation and controls the excessive mesh distortion that occurres due to sharp tool penetration. That is valid in case of modeling feed value large with respect to cutting tool radius.
3) Very low damage criterion for elements susceptible to excessive distortion can solve meshing problems related to tool penetration in workpiece. For instance, elements towards tool-point can be defined with very low damage value to cause fast deletion before any excessive distortion. It should be noted that these element must represent only small portion in workpiece not to afflict the accuracy of results.
4) LAG and ALE approaches can simulate the orthogonal cutting process and predict the cutting forces but with different accuracy. As maximum errors for LAG and ALE models were 14.4% and 5.8 %, while the minimum errors were 3.6% and 0.14%, respectively.
5) Chip simulation is smoother in ALE model, but it is more reliable and closer to experiments in LAG model.
6) Adaptive meshing and remeshing rules in FEA can control the excessive mesh distortion in metal cutting simulation, as well they increases the accuracy and steadiness of ALE results rather than LAG results.
7) Using very thin area of dense mesh below the penetration line of tool tip, in workpiece model, could reduce the simulation time to 44% without conflicting the accuracy of results.

## Declarations

### Author contribution statement

H. A. Soliman, A. Y. Shash, T. M. El Hossainy & M. Abd-Rabou: Conceived and designed the experiments; Performed the experiments; Analyzed and interpreted the data; Contributed reagents, materials, analysis tools or data; Wrote the paper.

### Funding statement

This research did not receive any specific grant from funding agencies in the public, commercial, or not-for-profit sectors.

### Data availability statement

Data included in article/supplementary material/referenced in article.

### Declaration of interests statement

The authors declare no conflict of interest.

### Additional information

No additional information is available for this paper.
